# Endogenous IL-33 has no effect on the progression of fibrosis during experimental steatohepatitis

**DOI:** 10.18632/oncotarget.18335

**Published:** 2017-06-01

**Authors:** Philippe Vasseur, Sarah Dion, Aveline Filliol, Valentine Genet, Catherine Lucas-Clerc, Girard Jean-Philippe, Christine Silvain, Jean-Claude Lecron, Claire Piquet-Pellorce, Michel Samson

**Affiliations:** ^1^ Service d'Hépato-Gastroentérologie, Centre Hospitalier Nord Deux-Sèvres, Thouars, France; ^2^ Laboratoire Inflammation Tissus Epithéliaux et Cytokines, Université de Poitiers, Poitiers, France; ^3^ Institut National de la Santé et de la Recherche Médicale, Institut de Recherche Santé Environnement & Travail, Université de Rennes, Rennes, France; ^4^ Service de Biochimie, Centre Hospitalier Universitaire, Rennes, Université de Rennes, Rennes, France; ^5^ Institut de Pharmacologie et de Biologie Structurale, Centre National de la Recherche Scientifique, Université de Toulouse, Toulouse, France; ^6^ Service d'Hépato-Gastroentérologie, Centre Hospitalier Universitaire de Poitiers, Poitiers, France; ^7^ Service d'Immunologie et Inflammation, Centre Hospitalier Universitaire de Poitiers, Poitiers, France

**Keywords:** nonalcoholic steatohepatitis, NASH, nonalcoholic fatty liver disease, NAFLD, immune cells

## Abstract

Interleukin (IL)-33 has been recently reported to be strongly pro-fibrogenic in various models of liver disease. Our aim was to study the role of endogenous IL-33 in a diet-induced model of steatohepatitis. IL-33 deficient mice and wild type (WT) littermates received a high-fat diet (HFD), or a standard diet for 12 weeks. The HFD-induced steatohepatitis was associated with an upregulation of IL-33 transcripts and protein. An insulin tolerance test revealed lower systemic insulin sensitivity in IL-33-/—HFD mice than in WT-HFD mice. Nevertheless, IL-33 deficiency did not affect the severity of liver inflammation by histological and transcriptomic analyses, nor the quantity of liver fibrosis. Livers from HFD mice had more myeloid populations, markedly fewer NKT cells and higher proportion of ST2+ Treg cells and ST2+ type 2 innate lymphoid cells (ILC2), all unaffected by IL-33 deficiency. In conclusion, deficiency of endogenous IL-33 does not affect the evolution of experimental diet-induced steatohepatitis towards liver fibrosis.

## INTRODUCTION

Nonalcoholic Fatty Liver Disease (NAFLD) is a significant cause of mortality due to liver-related complications, *i.e*. cirrhosis and hepatocellular carcinoma, and its extrahepatic manifestations, such as cardiovascular diseases [1]. According to the “two-hit” hypothesis, the early stage of NAFLD is characterized by non-inflammatory hepatic steatosis, a consequence of metabolic syndrome and insulin resistance. Nonalcoholic steatohepatitis (NASH) then develops in response to inflammatory stimuli, such as lipotoxicity or bacterial translocation, leading to cell death and fibrosis [2]. The development of liver fibrosis during NAFLD is clinically serious and the degree of fibrosis is exponentially associated with the mortality [3]. However, no specific therapy is currently recommended to treat fibrosis in patient with NAFLD, apart from lifestyle modifications [4]. Cytokines are pivotal mediators of liver fibrogenesis and tightly control, directly or indirectly, the transition of quiescent hepatic stellate cells (HSC) to collagen-secreting myofibroblasts [5]. Among all cytokines involved in liver fibrogenesis, we have shown that interleukin (IL)-33 hepatic expression is upregulated in cirrhotic patients and in a toxic model of liver fibrosis [6], and sustained release of IL-33 from liver cells has been shown to promote severe fibrosis in mice [7]. IL-33 is a cytokine of the IL-1 family, expressed by epithelial cells (including hepatocytes) [8, 9], endothelial cells [10], and fibroblasts [6, 11]. At steady state, IL-33 is localized to the nucleus and acts as a transcriptional regulator [12], but acts as an alarmin when cell necrosis or cell injury occurs [13]. Indeed, IL-33 can be released and displays pleiotropic activities through its receptor, ST2, expressed by various cell types such as T helper (Th)2 lymphocytes, regulatory T cells (Treg cells), natural killer T (NKT) cells, or type 2 innate lymphoid cells (ILC2) [14]. Recently, Gao *et al*. demonstrated that administration of recombinant exogenous IL-33 aggravates liver fibrosis during experimental NAFLD [15]. This prompted us to study the implication of endogenous IL-33 during experimental NAFLD. IL-33 regulates adipogenesis and the inflammatory phenotype of adipose tissue macrophages [16]. Thus, we used a diet-induced model of steatohepatitis, where mice are not obese and do not display features of metabolic syndrome, to focus on the implication of IL-33 in liver pathology and bypass indirect activity on metabolic parameters [17, 18].

## RESULTS

### High-fat diet induces hepatic expression of IL-33 and ST2

**Figure 1:**
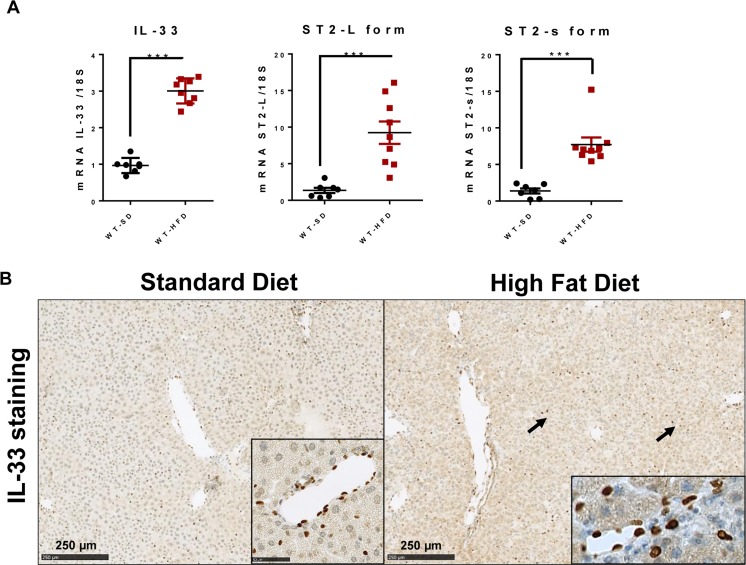
High-fat diet induces hepatic IL-33 expression Wild type mice received a HFD or a standard diet SD for 12 weeks. **A**. Relative mRNA expression of IL-33, and its membrane and soluble receptors ST2-L and ST2-s in livers from SD- and HFD-fed mice. The y-axis values represent the induction of each gene relative to control (SD-fed mice) after normalization using 18S. **B**. Immunolocalization of IL-33 in the livers of SD- and HFD-fed mice. Cellular staining is shown in the insets. Scale bars = 250 μm. Statistical analysis of the data was performed using the non-parametric Mann-Whitney U-test. Differences were considered to be significant for *p* < 0.05 and are indicated as follows: ***p < 0.001.

Wild-type mice were given the HFD or SD for 12 weeks. Livers from HFD mice showed upregulation of IL-33 transcripts, as well as its membrane receptor, ST2-L, and the soluble variant, ST2-s (Figure 1A). We detected constitutive IL-33 production by endothelial cells of SD mouse livers by immunohistochemical analysis. IL-33 production was upregulated in HFD mice and was mostly found in the perisinusoidal spaces (Figure 1B).

### IL-33 deficiency moderately affects systemic insulin sensitivity

**Figure 2:**
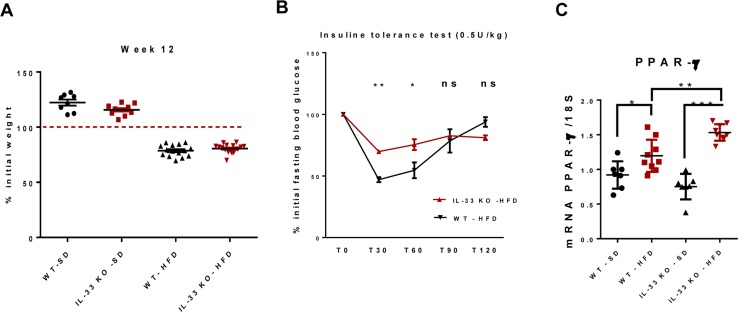
IL-33 deficiency moderately affects systemic insulin sensitivity **A**. Variation of body weight after 12 weeks of SD HFD in WT and IL-33 KO mice. **B**. Insulin tolerance test conducted at week 9 in IL-33 KO-HFD and WT-HFD mice. Mice received an *i.p*. injection of 0.5U/kg insulin after a four-hour fast. Blood glucose concentrations were measured 0, 30, 60 and 120 minutes after injection. **C**. Relative mRNA transcript levels of the lipogenic factor PPAR-γ in the liver. The y-axis values represent the induction of PPAR-γ relative to control (SD-fed mice) after normalization using 18S. Statistical analysis of the data was performed using the non-parametric Mann-Whitney U-test. Differences were considered to be significant for p < 0.05 and are indicated as follows: *p < 0.05, **p < 0.01, ***p < 0.001, ns: non-significant.

Mice fed the HFD were non-obese, as expected. The weight of IL-33 KO mice on either diet was similar to that of their littermates (Figure 2A). We studied systemic insulin sensitivity by intraperitoneally injecting 0.5U/kg insulin. WT-SD and WT-HFD mice had similar profiles of insulin tolerance (data not shown), whereas IL-33 KO-HFD mice were less sensitive to insulin than WT-HFD mice (Figure 2B). We studied the hepatic expression of PPAR-γ as adipogenesis is tightly associated with insulin signaling. IL-33 deficiency further increased hepatic overexpression of PPAR-γ already induced by the HFD (Figure 2C).

### IL-33 deficiency does not affect HFD-induced liver inflammation nor fibrosis

**Figure 3:**
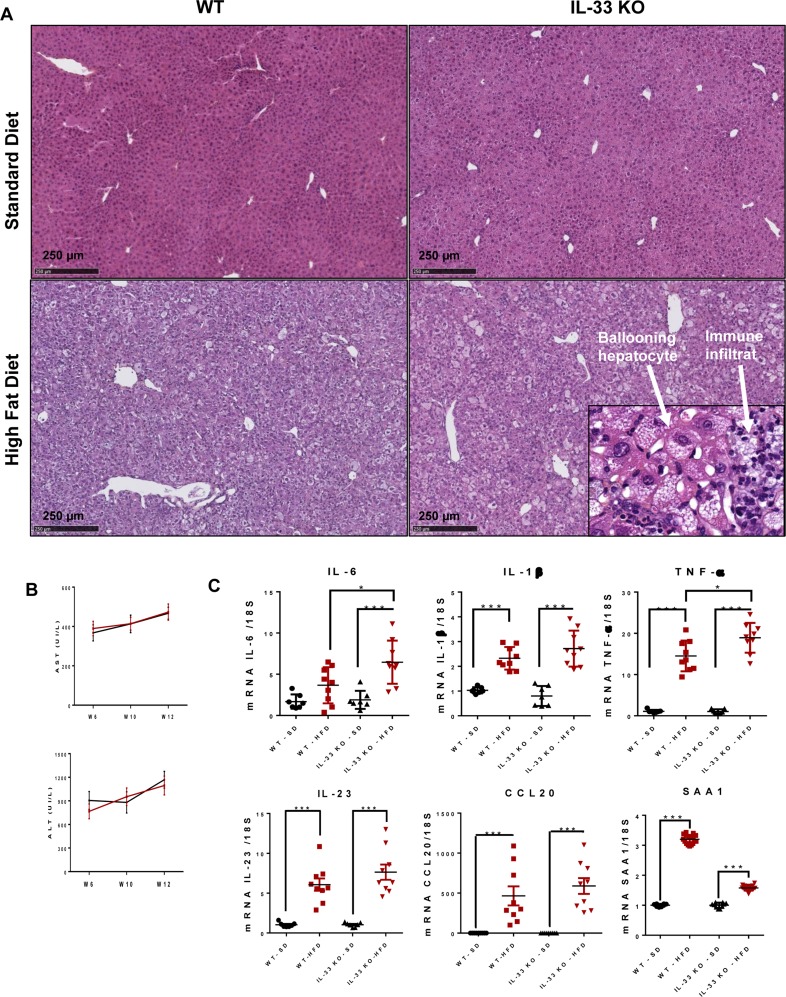
IL-33 deficiency does not alter the inflammatory features of steatohepatitis in mice fed the high-fat diet **A**. Representative liver histology (HE coloration) for IL-33 KO mice and WT littermates fed HFD or SD. Ballooning hepatocytes are shown in the insets. Scale bar = 250 μm. **B**. Levels of serum ALT and AST (UI/L) at weeks 6, 10 and 12 in WT and IL-33 KO mice fed a HFD. **C**. Relative mRNA transcript levels of IL-6, IL-1β, TNF-α, IL-23, CCL20 and SAA in livers. The y-axis values represent the induction of each gene relative to control (SD-fed WT mice) after normalization using 18S. Statistical analysis of the data was performed using the non-parametric Mann-Whitney U-test. Differences were considered to be significant for *p* < 0.05 and are indicated as follows: *p < 0.05, ***p < 0.001, ns: non-significant.

IL-33 KO-HFD mice did not show an exacerbation of hepatocyte steatosis after 12 weeks of feeding, by semi-quantitative analysis of H&E stained livers (Figure 3A). The HFD induced steatohepatitis characterized by major macro- and micro-vacuolar steatosis, lobular infiltrates, and frequent ballooning hepatocytes, a required feature for the diagnosis of NASH in humans [19]. IL-33 deficiency did not affect the severity of steatohepatitis at the histological level: the degree of steatosis, and the frequency of lobular infiltrates and ballooning hepatocytes was similar between WT-HFD and IL-33 KO-HFD mice (Figure 3A). Serum transaminases activity, reflecting hepatocyte injury, increased when mice were fed with the HFD but did not differ at any time point between WT and IL-33 KO mice (Figure 3B). In accordance with these results, the HFD-induced overexpression of the Th17-related cytokines IL-23 and CCL20 was not altered by IL-33 deficiency. However, liver IL-6 and TNF-α transcript levels were higher in IL-33 KO-HFD mice than WT-HFD mice. Corroborating this latter observation, SAA protein transcripts, an acute phase protein mainly induced by pro-inflammatory cytokines such as IL-6, IL-1 or TNF-α, were overexpressed in IL-33 KO-HFD mice compared to WT-HFD mice (Figure 3C).

**Figure 4:**
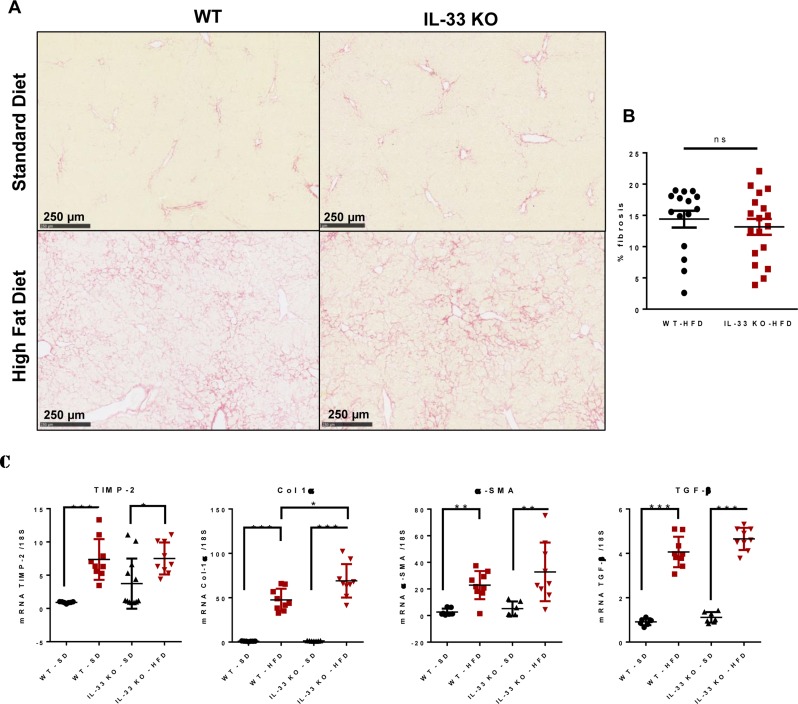
Evolution of diet-induced steatohepatitis towards fibrosis is not affected by IL-33 deficiency **A**. Representative liver sections stained with Sirius red from WT and IL-33 KO mice fed either SD or HFD. Scale bar = 250μm. **B**. Area of fibrosis according to the percentage of total liver sections stained with Sirius red in WT and IL-33 KO mice fed the HFD. **C**. Relative mRNA transcript levels of the fibrosis markers TIMP-2, Col 1α, α-SMA, and TGF-β in liver. The y-axis values represent the induction of each gene relative to control (SD-fed WT mice) after normalization using 18S. Statistical analysis of the data was performed using the non-parametric Mann-Whitney U-test. Differences were considered to be significant for *p* < 0.05 and are indicated as follows: *p < 0.05, **p < 0.01, ***p < 0.001, ns: non-significant.

The inflammatory phenomena observed in HFD-fed mice progressed towards significant fibrosis. Sirius red staining showed periportal and perisinusoidal fibrosis with a “chicken wire” pattern, as observed in the human disease [20]. There was no difference in the severity of fibrosis between WT-HFD mice and IL-33 KO-HFD mice [14.4 % (*n* = 15) *vs* 13.1 % (*n* = 18), *p* = 0.477)] (Figure 4A). Liver fibrosis is the consequence of excessive collagen deposition and inhibition of collagen degradation. The expression of Col-1 α1, α-SMA, TGF-β1 and TIMP-2 transcripts was much higher in both IL-33 KO and WT mice fed the HFD than those fed the SD, but, IL-33 deficiency did not alter liver expression of TIMP-2, in agreement with the Sirius red staining. Nonetheless, IL-33 KO-HFD mice had higher Col-1 α1 transcript levels than WT-HFD mice. Liver transcript levels of α-SMA and TGF-β1, two markers of activated HSC, did not differ between the two groups (Figure 4B).

### High-fat diet induces significant changes in immune composition of the liver

**Figure 5:**
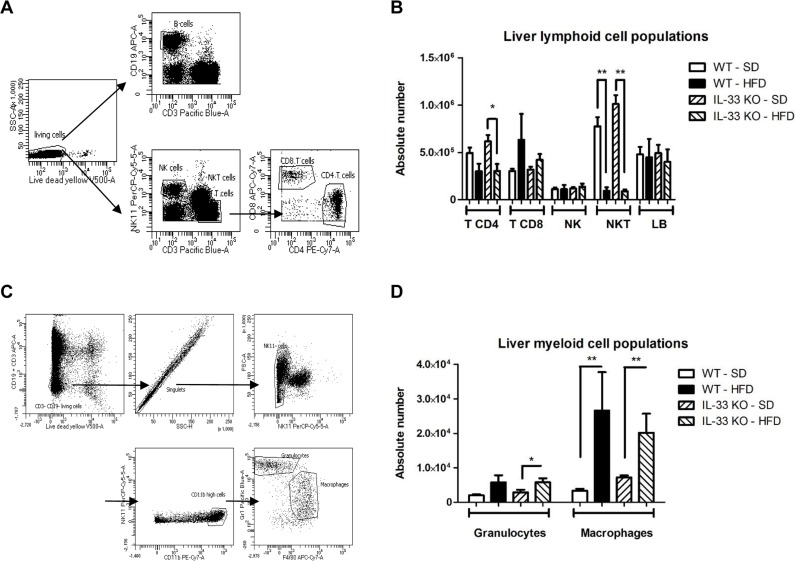
High-fat diet induces changes in immune composition of the liver **A**. Gating strategy for liver lymphoid cell populations. B cells (CD19^+^CD3^−^), NK cells (NK1.1^+^CD3^−^), NKT cells (NK1.1^+^CD3^+^), CD8 T cells (CD3^+^NK1.1^−^CD8^+^) and CD4 T cells (CD3^+^NK1.1^−^CD4^+^) were first gated for singlets and on a viability marker. **B**. Absolute numbers of the various lymphoid immune cells in livers from WT and IL-33 KO mice fed a SD or HFD. **C**. Gating strategy for liver myeloid cell populations. Granulocytes (CD11b^high^Gr1^+^F4/80^−^) and macrophages (CD11b^high^Gr1^−^F4/80^+^) were first gated for singlets and on a viability marker. **D**. Absolute numbers of the various myeloid immune cells in livers from WT and IL-33 KO mice fed a SD or a HFD. Statistical analysis of the data was performed using the non-parametric Mann-Whitney U-test. Differences were considered to be significant for *p* < 0.05 and are indicated as follows: *p < 0.05, **p < 0.01.

We characterized the lymphoid and myeloid composition of the livers by flow cytometry analysis of living cells after 12 weeks of feeding (Figure 5A and 5C). The principal effect of the HFD on lymphoid cell composition was marked decrease in the absolute number of NKT cells (Figure 5B) including CD4 positive and double negative CD4 and CD8 NKT cells (Supplemental Figure 1); deficiency of endogenous IL-33 had no effect on this reduction (Figure 5B). At the same time, the HFD tended to decrease the number of CD4^+^ T cells and increase the number of CD8^+^ T cells in WT and IL-33 KO mice (Figure 5B). Analysis of the lymphoid activation marker CD69 showed that the HFD induced significant activation of CD8^+^ T cells (*p* < 0.01) (Supplemental Figure 2), which was unaffected by IL-33 deficiency. Analysis of myeloid populations showed that steatohepatitis was associated with an increase in the number of CD11b^high^ Gr1^+^ granulocytes and CD11b^high^ F4/80^+^ macrophages, as expected, and that was unaffected by IL-33 deficiency (Figure 5D).

### IL-33 deficiency does not alter liver Treg ST2^+^ and ILC2 populations in mice with steatohepatitis

**Figure 6:**
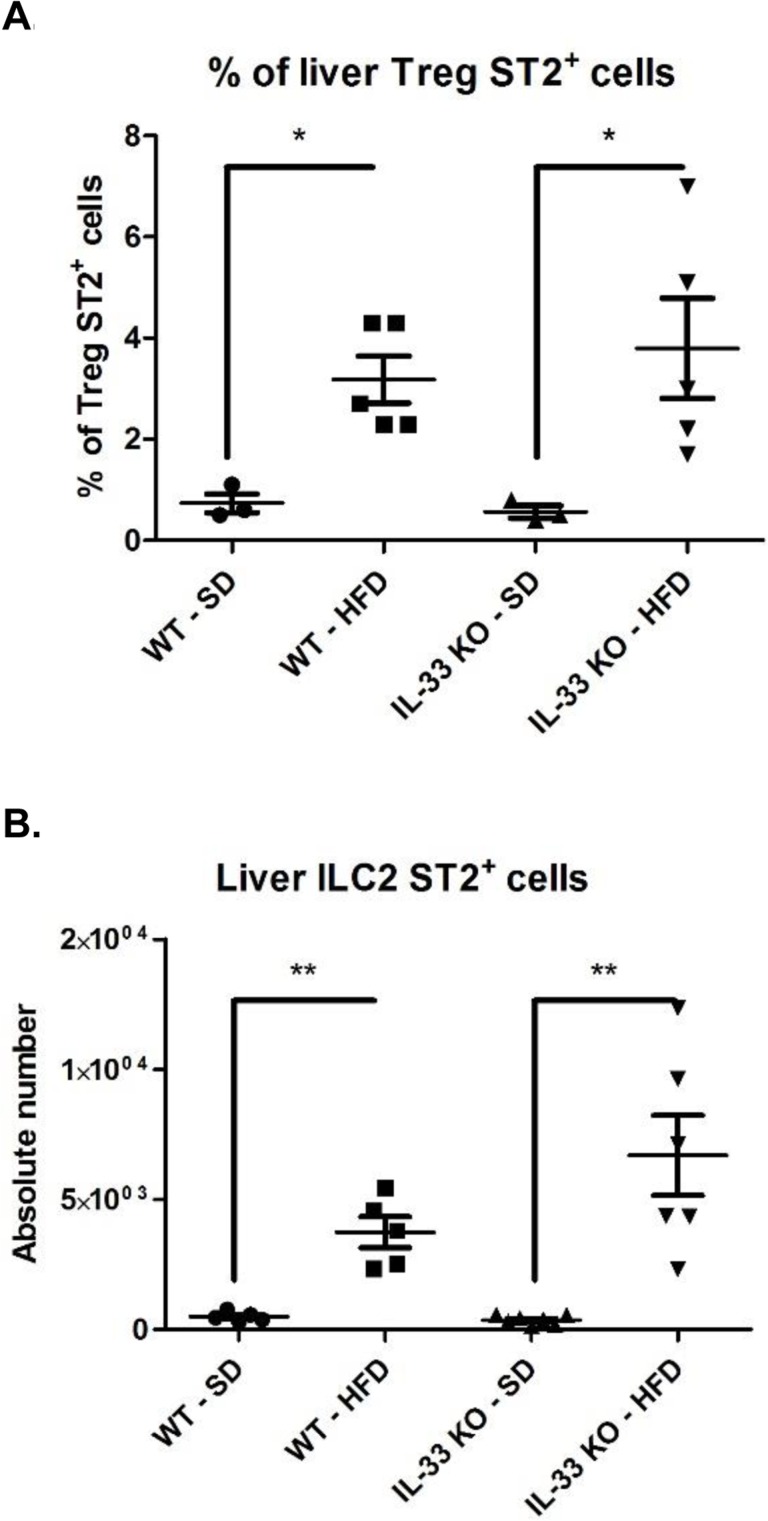
The increased proportion of liver ST2+ T reg and ST2+ type 2 innate lymphoid cells induced by the high-fat diet is exacerbated by IL-33 deficiency **A**. Results are expressed as the means ± SEM of the percentage for liver Treg ST2^+^. **B**. Results are expressed as the means ± SEM of absolute numbers for liver ILC2 ST2^+^. Statistical analysis of the data was performed using the non-parametric Mann-Whitney U-test. Differences were considered to be significant for *p* < 0.05 and are indicated as follows: *p < 0.05, **p < 0.01.

Finally, we focused on the immune cell targets of IL-33, *i.e*. ST2-expressing cells; particularly Treg ST2^+^ cells, which display the highest expression of the IL-33 receptor in experimental acute hepatitis [21], and ILC2, which are essential downstream effectors in IL-33-mediated liver fibrosis [7]. After gating on CD3^+^ NK1.1^−^ and CD4^+^ cells, we characterized Treg cells (FoxP3^+^) based on ST2 expression. The HFD upregulated the expression of ST2 by Treg cells and IL-33 deficiency had no significant impact on the proportion of Treg ST2^+^ cells among CD4^+^ T lymphocytes (Figure 6A). Because ILC2 are lineage negative cells (CD11b^−^, Gr1^−^, CD3ε, CD4^−^, CD8α^−^, CD19^−^, FcεR1^−^ and NK1.1^−^), we used this gating to characterize ILC2 in WT-HFD and IL-33 KO-HFD mice. The HFD increased the proportion of ST2^+^ ILC2, which was unaffected by IL-33 deficiency (Figure 6B).

## DISCUSSION

Type-2 immune cells, such as ILC2 or Th2 lymphocytes, are critically involved in fibroproliferative diseases [22]. Aside from the secretion of type-2 cytokines, these cells also express ST2, the receptor for IL-33 [23]. IL-33 is pro-fibrogenic, as its hepatic expression is sufficient to drive severe liver fibrosis through ILC2-derived IL-13; which is further demonstrated by a strong reduction of experimentally-induced liver fibrosis in mice lacking *IL-33* [7]. Systemic administration of recombinant exogenous IL-33 has been shown to exert a dual activity in two murine models of NASH. Treatment with rIL-33 attenuated weight gain, systemic insulin resistance and consequent hepatic steatosis in mice fed with a HFD, but exacerbated liver fibrosis in mice exposed to the HFD or a methionine choline-deficient (MCD) diet [15]. Consistent with these results, we observed that mice lacking *IL-33* and fed a HFD were less sensitive to insulin and displayed hepatic overexpression of the lipogenic marker PPAR-γ. IL-33 can improve metabolic parameters through restoration of ST2^+^ Treg cells and accumulation of anti-inflammatory (M2) macrophages in the adipose tissue of obese mice, which are associated with an improvement of obesity-induced insulin resistance [24, 35]. Our results concerning the severity of liver fibrosis are in disagreement with those of the study of Gao *et al*. [15]. Indeed, liver transcripts of IL-33 were overexpressed in our NASH model, but IL-33 deficiency did not improve the liver disease. Fibrosis and inflammation, as shown by histological analysis and quantification of fibrogenic markers, were similar in WT and IL-33 KO mice. We measured serum transaminases activity to exclude delayed effects of IL-33 deficiency, but they were equivalent in both groups at all time points. These conflicting results may be due to several factors. First, the mice received large amounts of rIL-33 in the Gao *et al.* study (up to 40 *i.p*. injections of 1 μg rIL-33), which may not correspond to physiological concentrations [15], and the route of IL-33 injection (i.p.) did not specifically target the liver but all ST2^+^ immune cells, starting with the cells of the peritoneal cavity. Second, we used a diet-induced model of NASH which was different from that of Gao *et al*. They used a “standard” HFD, in which 58% of the energy comes from fat [15], which induces obesity and systemic insulin resistance, but does not reproduce the human disease in mice; hepatocyte ballooning, the histological hallmark of NASH, is infrequent in this model and the fibrosis is mild [26]. The addition of cholesterol and cholate to the high fat component, in our study, leads to significant inflammatory changes with hepatocellular ballooning and severe fibrosis [17]. The mice are not obese and there is no expansion of adipose tissue with our diet, but they display hepatocellular insulin resistance, a central event in the pathogenesis of NASH [27]. Third, in contrast to the injection of rIL-33, which only acts *via* interaction with ST2, IL-33 KO mice also lack the intracellular form that regulates the expression of various gene. For example, nuclear IL-33 can sequester NF-κB and inhibit the expression of its target genes such as TNF-α [28], and it can repress IL-6 expression [29]. Indeed, we observed that IL-33 deficiency exacerbated the HFD-induced hepatic expressions of IL-6, TNF-α and their target gene SAA. Thus, it is possible that the extracellular form of IL-33 triggers fibrosis, as IL-33-induced liver fibrosis is totally reversed in ST2 deficient mice, whereas the hydrodynamic delivery of intracellular IL-33 in IL-33 KO mice does not induce liver disease [7]. In summary, the pro-fibrotic activity of extracellular IL-33 and anti-inflammatory properties of nuclear IL-33 may compensate each other and explain, in part, our results. In the same way, it was reported that deficiency of the endogenously produced IL-33 and its receptor ST2 did not impact the development of atherosclerosis in ApoE-deficient mice [30] while the administration of exogenous IL-33 reduces the development of atherosclerosis [31].

Our results substantially differ from those that describe the activity of IL-33 in models of acute hepatitis [32]. For example, during concanavalin A (ConA)-induced hepatitis, IL-33 exerts protective effects and is mainly expressed by hepatocytes as a consequence of interaction with NKT cells and through the TRAIL pathway [8, 9]. In the carbon tetrachloride-induced fibrosing hepatitis model, IL-33 expression is induced in sinusoidal cells and HSC [6], consistent with a perisinusoidal immunostaining in our study.

We observed that IL-33 deficiency did not alter the proportion of ST2^+^ Treg cells and ST2^+^ ILC2 in mice with steatohepatitis. Our results suggest that IL-33 is not necessary for the maintenance of these two cellular populations during chronic liver injury, in contrast to previously published data indicating that IL-33 displays a strong survival and proliferative effect *in vitro* on Treg cells [21, 33] and is able to expand liver ILC2. However, in the setting of ConA-induced acute hepatitis, IL-33 exerts a dual activity through its cellular targets. The resolution of hepatitis in rIL-33-treated mice in this model is associated with an expansion of liver ST2^+^ Treg cells, whereas rIL-33-induced ILC2 exerts deleterious effects in this model [34].

Finally, consistent with other models of HFD-induced steatohepatitis [35–37], we show a marked decrease in the number of hepatic NKT cells in NASH mice, that is unaffected by IL-33 deficiency. This may be related to activation-induced cell death of NKT cells by lipids, as they recognize the CD1d molecule, which binds lipid and glycolipid antigens. In the present work, the HFD has been shown to increase the hepatic concentration of free fatty acids [17], and saturated fatty acids enhance the activation and subsequent apoptosis of NKT cells through CD1d [37]. However, the role of NKT cells during experimental NAFLD remains unclear [38, 39].

In conclusion, IL-33 deficiency in mice does not lessen liver fibrosis during diet-induced steatohepatitis, in contrast to previous studies indicating a deleterious role of exogenous IL-33 in chronic liver injury and experimental NAFLD.

## MATERIALS AND METHODS

### Animals

Eight- to ten-week-old male IL-33 knockout (IL-33 KO) C57BL/6 mice (provided by Dr Jean-Philippe Girard) [40] and age-matched wild type (WT) littermates were given a standard diet (SD) (5001, LabDiet, St. Louis, MO) or a high-fat diet (HFD) enriched in cholate, described elsewhere [41], *ad libitum* for 12 weeks. All mice were reared in specific pathogen-free conditions at the local animal house facilities. The study was conducted in accordance with French law and institutional guidelines for animal welfare. All efforts were made to minimize suffering and the number of animals involved. The protocol was approved by the “Comité Rennais d'Ethique en matière d'Expérimentation Animale”, the local ethics committee accredited by the French Ministry of Research and Higher Education (protocol agreement number: R-2012-CPP-Ol, researcher agreement for M. Samson #35-96 and C. Piquet-Pellorce #35-82).

### Histological, immunohistochemical and biochemical analyses

Liver pathology was characterized from H&E stained sections based on the semi-quantitative NAFLD activity score (NAS) which evaluates the degree of steatosis, lobular infiltrates and hepatocytes ballooning [42]. Liver fibrosis was assessed by Sirius red coloration, and the stained area calculated from the total liver surface using NIS-Elements software (Nikon). Immunolocalisation of IL-33 was performed using a primary goat IgG anti-mouse-IL-33 antibody (R&D Systems) and a secondary HRP-conjugated rabbit anti-goat antibody (Dako, USA) with hematoxylin counterstaining in a Ventana machine (Ventana Medical Systems, Inc. USA). Serum biochemical analyses of transaminases levels (AST/ALT) were performed as described previously [9].

### RNA isolation and RT-qPCR

Total RNA was extracted from mouse liver pieces using TRIzol Reagent (Invitrogen, Carlsbad, CA). First-strand cDNA was produced using SuperScriptTM II Reverse Transcriptase (Invitrogen). Real-time qPCR was performed using the fluorescent dye SYBR Green with the double-strand specific dye SYBRs Green system (Applied Biosystems) and the 7300 sequence detection system ABI Prism sequence detector (Applied Biosystems). Total cDNA (30 ng) was used as a template for amplification with the specific primer pair (Table 1) used at a final concentration 300nM. Each measurement was performed in triplicate. The mRNA level of mouse IL-33, ST2-L, ST2-s, peroxisome proliferator-activated receptor (PPAR)-γ, IL-6, IL-1β, tumor necrosis factor (TNF)-α, IL-23, C-C motif chemokine ligand 20 (CCL20), serum amyloid A (SAA) protein, collagen type 1 type α1 (Col-1α), α-smooth muscle actin (α-SMA), transforming growth factor (TGF)-β1, and tissue inhibitor metalloproteinase (TIMP)-2 were normalized to the mRNA expression of ubiquitous house-keeping gene 18S.

**Table 1:** Sequences of specific primers used for RT-qPCR

Genes	Forward	Reverse
IL-33	5′ATGGGAAGAAGCTGATGGTG 3′	5′CCGAGGACTTTTTGTGAAGG3′
ST2L	5′ATTCAGGGGACCATCAAGTG 3′	5′CGTCTTGGAGGCTCTTTCTG 3′
ST2S	5′CGTGTCCAACAATTGACCTG 3′	5′CTGAACCTTGGCTCTTGGAG3′
PPAR-γ	5′CTGATGCACTGCCTATGAGC 3′	5′GGGTCAGCTCTTGTGAATGG3′
IL-1β	5′GGACCCCAAAGATGAAGG 3′	5′GTAGCTGCCACAGCTTCTCC 3′
TNF-α	5′CTGGTGACCCTGTTGTTGG 3′	5′TGAGAGGCTAGAGGGTGAGG 3′
IL-23	5′CAGGGCTGAGACTACAAACG 3′	5′GGCTGCCAGTTTCTTTTACC 3′
CCL20	5′TCTGCTCTTCCTTGCTTTGG 3′	5′TCACCCAGTTCTGCTTTGG3′
COL1-α	5′GCTCCTGCTCCTCTTAGGG 3′	5′GCAGAAAGCACAGCACTCG 3′
α-SMA	5′ GGAATCCTGTGAAGCAGCTC 3′	5′CAGAGCCATTGTCACACACC 3′
TGF-β	5′CACCATCCATGACATGAACC 3′	5′CAGAAGTTGGCATGGTAGCC 3′
TIMP2	5′ATAGATGTCATTCCCGGAAT3′	5′TGCAATGCAATTTCCAGGAC 3′

### Insulin tolerance test

Mice at week 12 received an *i.p*. injection of 0.5UI/kg insulin (Humalog, Lilly, Indianapolis, IN) after a four-hour fast. The blood glucose concentration was measured from the tail vein, 0, 30, 60, 90, and 120 min after injection, with a calibrated blood glucose monitoring system (FreeStyle Easy, Abbott, Chicago, IL).

### Isolation of liver immune cells and flow cytometry

The liver immune cells were isolated as previously described [43, 44], with a viability > 95%. Liver cells were resuspended in staining buffer (10% FCS in PBS) and incubated with anti-CD16/32 antibody (BD Pharmingen) to block non-specific binding. The cells were then labeled with the appropriate fluorochrome-conjugated antibodies/reagents (BD Pharmingen and eBioscience): orange LIVE/DEAD, anti-CD3-PacificBlue (clone 500-A2), anti-CD3-APC (clone 145.2.C11), anti-CD4-PE-Cy7 (clone RM4-5), anti-CD8-APC-Cy7 (clone 53-6.7), anti-CD25-PEeFluor610 (clone PC61.5 or clone 3C7), anti-NK1.1-PerCP-Cy-5.5 (clone PK136), anti-ST2-PE (clone RMST2-33), anti-CD11b-PE Cy^TM^7 (clone M1/70), anti-Gr1-V450-Ly.6G/C (clone RB6-8C5) and CD19-APC (clone 1D3), anti-F4/80-APCeFluor780 (clone B8M), anti-Fc-εRIα-FITC (clone MAR-1), and anti-CD69-PE (clone H1.2.F3). After membrane staining, intranuclear staining was carried out with the Foxp3-Alexa488 antibody (clone MF23), according to the manufacturer's instructions. The stained cells were analyzed on a FACSAria^TM^ II flow cytometer with BD FACSDiva software (BD Bioscience) and the data analyzed using BD FACSDiva software (BD Bioscience). Doublets and dead cells were excluded on the basis of forward/side scatter and LIVE/DEAD labeling, respectively. We calculated the percentage of each immune cell population, by considering the sum of events of

all immune cell populations analyzed (sum of T, NK, NKT, B cells or myeloid cells) as 100% of the total immune cells. The absolute number in each immune cell population was calculated by multiplying the percentage of each population by the total number of immune cells.

### Statistical analysis

The results shown are representative of two independent experiments and are expressed as the means ± SEM for each group of mice (3 to 10 mice per group from two independent experiments). We used the nonparametric Mann-Whitney *U* test, as implemented in GraphPad Prism5 software. Differences were considered to be significant for *p* < 0.05 and are indicated as follows: * *p* < 0.05, **p < 0.01 and ***p < 0.001.

## SUPPLEMENTARY MATERIALS FIGURES



## Abbreviations

AST: aspartate aminotransferase
ALT: alanine aminotransferase
knockout: KO or −/−
NASH: nonalcoholic steatohepatitis
NAFLD: nonalcoholic fatty liver Disease
HFD: high-fat diet
Treg cells: regulatory T cells
NKT: natural killer T cells.
