# Supplementary material for: Endogenous IL-33 has no effect on the progression of fibrosis during experimental steatohepatitis
Source: Oncotarget. 2017 Jun 1;8(30):48563–74. doi: 10.18632/oncotarget.18335 (PMC5564708; doi:10.18632/oncotarget.18335)
Supplement: Supplementary file 1 [file oncotarget-08-48563-s001.pdf]

Endogenous IL-33 has no effect on the progression of fibrosis during experimental steatohepatitis

Supplementary Material

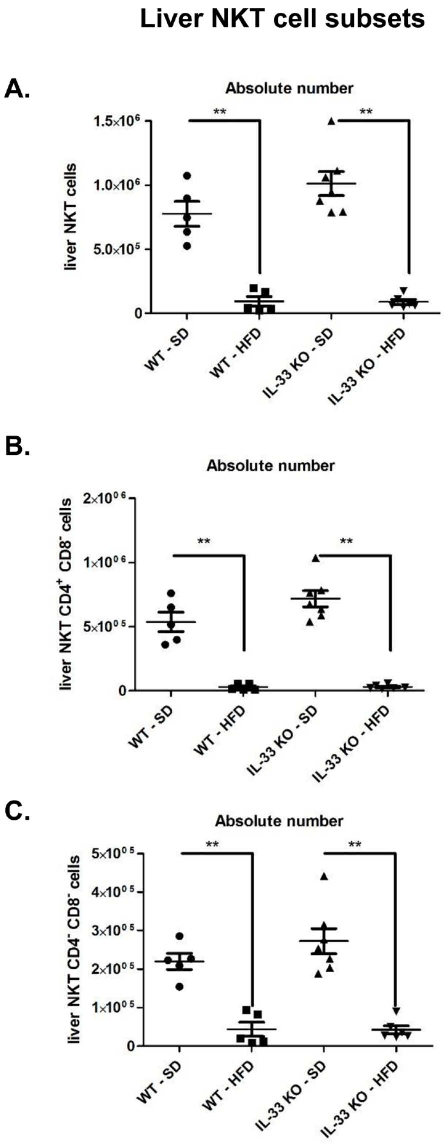

Supplemental Figure 1: High-fat diet induces significant changes in NKT cell subsets in wild-type and IL-33 KO mice. The absolute numbers of the various NKT cells subsets in livers from mice were evaluated by flow cytometry. (A) Total NKT cells, (B) the NKT $CD4^+$ $CD8^-$ cell subset and (C) the NKT $CD4^-$ $CD8^-$ cell subset. NKT cells are defined as $CD3^+$ $NK1.1^+$, and CD4/CD8 staining was used to define the NKT cell subsets. Statistical analysis of the data was performed using the non-parametric Mann–Whitney U-test. Differences were considered to be significant for $p < 0.05$ and are indicated as follows: *$p < 0.05$, **$p < 0.01$, ***$p < 0.001$.

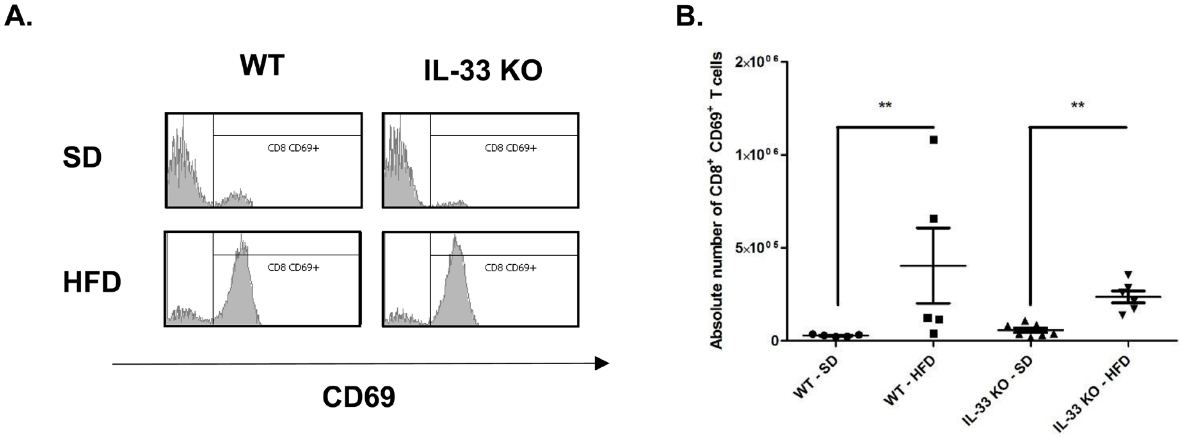

Supplemental Figure 2: The high-fat diet induces significant activation of CD4 and CD8 T cell subsets in wild-type and IL-33 KO mice. The activation level of the various immune cells subsets in liver from mice were evaluated by flow cytometry and the activation level as defined by CD69 staining. (A) Representative FACS histogram of CD69 staining used to define the numbers of activated CD8 cell found in livers. (B) Activation of CD8 T cell subsets. The results are expressed as the means ± SEM of absolute numbers for each immune population subset. Statistical analysis of the data was performed by using the non-parametric Mann–Whitney U-test. Differences were considered to be significant for $p < 0.05$ and are indicated as follows: **$p < 0.01$.
